# Development and validation of a prognostic nomogram for neuroendocrine prostate cancer, based on the SEER database

**DOI:** 10.3389/fsurg.2023.1110040

**Published:** 2023-03-10

**Authors:** Siming Chen, Kangping Xiong, Jiageng Shi, Shijie Yao, Gang Wang, Kaiyu Qian, Xinghuan Wang

**Affiliations:** ^1^ Department of Urology, Zhongnan Hospital of Wuhan University, Wuhan, China; ^2^ Department of Gynecological Oncology, Zhongnan Hospital of Wuhan University, Wuhan, China; ^3^ Department of Biological Repositories, Zhongnan Hospital of Wuhan University, Wuhan, China; ^4^ Laboratory of Precision Medicine, Zhongnan Hospital of Wuhan University, Wuhan, China; ^5^ Wuhan Research Center for Infectious Diseases and Cancer, Chinese Academy of Medical Sciences, Wuhan, China; ^6^ Medical Research Institute, Wuhan University, Wuhan, China

**Keywords:** SEER, neuroendocrine prostate cancer, nomogram, prognosis, overall survival, cancer-specific survival

## Abstract

**Background:**

The tumor biology of neuroendocrine prostate cancer (NEPC) is different from that of ordinary prostate cancer, herefore, existing clinical prognosis models for prostate cancer patients are unsuitable for NEPC. The specialized individual situation assessment and clinical decision-making tools for NEPC patients are urgently needed. This study aimed to develop a valid NEPC prognostic nomogram and risk stratification model to predict risk associated with patient outcomes.

**Methods:**

We collected 340 de-novo NEPC patients from the SEER database, and randomly selected 240 of them as the training set and the remaining 100 as the validation set. Cox regression model was used to screen for risk factors affecting overall survival (OS) and cancer-specific survival (CSS) and construct a corresponding nomogram. The receiver operating characteristic (ROC) curves, calibration curves, C-indexes, and decision curve analysis (DCA) curves are used to verify and calibrate nomograms.

**Results:**

NEPC prognosis nomograms were constructed by integrating independent risk factors. The C-indexes, ROC curves, calibration curves, and DCA curves revealed excellent prediction accuracy of the prognostic nomogram. Furthermore, we demonstrated that NEPC patients in the high-risk group had significantly lower OS and CSS than those in the low-risk group with risk scores calculated from nomograms.

**Conclusions:**

The nomogram established in this research has the potential to be applied to the clinic to evaluate the prognosis of NEPC patients and support corresponding clinical decision-making.

## Introduction

Prostate cancer (PCa) is one of the most common malignant tumors in men worldwide, ranking the first in morbidity and the second in mortality among men in the United States (1). In recent years, the incidence of prostate cancer in China has also continued to rise. As a subspecies of hormone-refractory prostate cancer, neuroendocrine prostate cancer (NEPC) is highly aggressive, lacks effective treatments and has a poor prognosis. In addition, NEPC patients have the following clinical characteristics: rapid deterioration and often accompanied by visceral metastasis and lytic bone lesions, obvious prostate hypertrophy, but low prostate-specific antigen levels (2). NEPC can be diagnosed after conventional adenocarcinoma therapy or as a de-novo entity. The former is mainly due to the inevitable development of castration-resistant prostate cancer after androgen deprivation therapy (ADT), and further expression of neuroendocrine markers to develop NEPC (2). However, the rate of neuroendocrine (NE) differentiation in prostate cancer after ADT is difficult to assess correctly, and many patients die from PCa without biopsy confirmation of NE differentiation, so cases are limited (3). De-novo NEPC is a rare and highly malignant disease that can be effectively diagnosed by biopsy (3). Based on this, we hope to study the prognostic factors of de-novo NEPC based on SEER, a database with a large sample size.

The tumor biology of NEPC is different from that of ordinary prostate cancer, which means that traditional prostate cancer treatments have limited efficacy in treating NEPC, so the existing clinical prognosis models for prostate cancer patients are not suitable for NEPC. We need to establish specialized individual situation assessment and clinical decision-making tools for NEPC patients.

Nomograms could integrate TMN staging and other key prognostic factors to assess patient prognosis, overall survival (OS), and cancer-specific survival (CSS). Nomograms are widely used to assess many types of cancers such as carcinoma of urinary bladder, inflammatory breast cancer, Non-Small-Cell lung cancer, and duodenal adenocarcinoma (4–7). It supports clinical decision making by predicting the prognosis of patients more accurately.

It is worth noting that although nomograms studies on prostate cancer have been reported, the independent prognostic factors of de-novo NEPC and ordinary prostate cancer are significantly different, we urgently need to establish a new prognostic model for de-novo NEPC instead of ordinary prostate cancer (8). Based on the SEER database, a de-novo NEPC-targeting nomogram prediction model was constructed to predict the OS and CSS of NEPC at 1-, 3-, and 5- year. This is the first nomogram model for assessing the patient prognosis of de-novo NEPC.

## Materials and methods

### Data collection and ethical statement

All data in our research were derived from the SEER database (https://seer.cancer.gov/). After obtaining the license, we collected information on the confirmed de-novo NEPC patients registered in the SEER database from 2010 to 2015. Since SEER is a public database with anonymous data, this study was waived from ethical review. By using the ICD-O-3 site code: C61.9 in combination with the histopathological code of neuroendocrine carcinoma: 8,012, 8,013, 8,020, 8,021, 8,041, 8,042, 8,240, 8,246 to screen NEPC cases. Inclusion criteria: (1) pathological diagnosis of pure NEPC; (2) de-novo NEPC; (3) Age ≥18; (4) The years of diagnosis were 2010–2015. Exclusion criteria: (1) absence of survival data; (2) non-primary tumors; (3) Cases recorded as T0; (4) patients with NE differentiation.

### Research factors

The patient's race, gender, marital status, age at diagnosis, AJCC TNM stage, treatment plan (surgery, radiotherapy, and chemotherapy), distant metastasis (bone, brain, liver, lung), and survival status and time were collected. The X-tile version 3.6.1 was used to determine the optimal threshold for age and nomogram points. The OS and CSS were identified as the study endpoints. OS was defined as the time from the NEPC diagnosis date to the date of death (event occurred) or last contact (censor). CSS was defined as the time from the NEPC diagnosis date to the date of death due to NEPC (event occurred) or last contact (censor).

### Statistical analysis

SPSS 26.0 software was used for data analysis, and the chi-square test or Fisher's exact test was used to compare categorical variables. Univariate and multivariate analyses were performed using Cox proportional hazards regression models to screen independent risk factors for OS and CSS in NEPC patients. Variables with significant differences in multivariate analysis were considered prognostic variables affecting patient survival, and a nomogram was drawn. The accuracy of the nomogram was assessed by C-index, calibration curve, DAC curve, ROC curve, and their corresponding AUC. Survival analysis between groups was performed using the Kaplan-Meier method and log-rank test. R 3.4.3 software is used to construct nomogram and generate ROC curve, calibration curve and DCA curve. *P* < 0.05 was considered significant difference.

## Results

### Characteristics of patients

**Figure 1:**
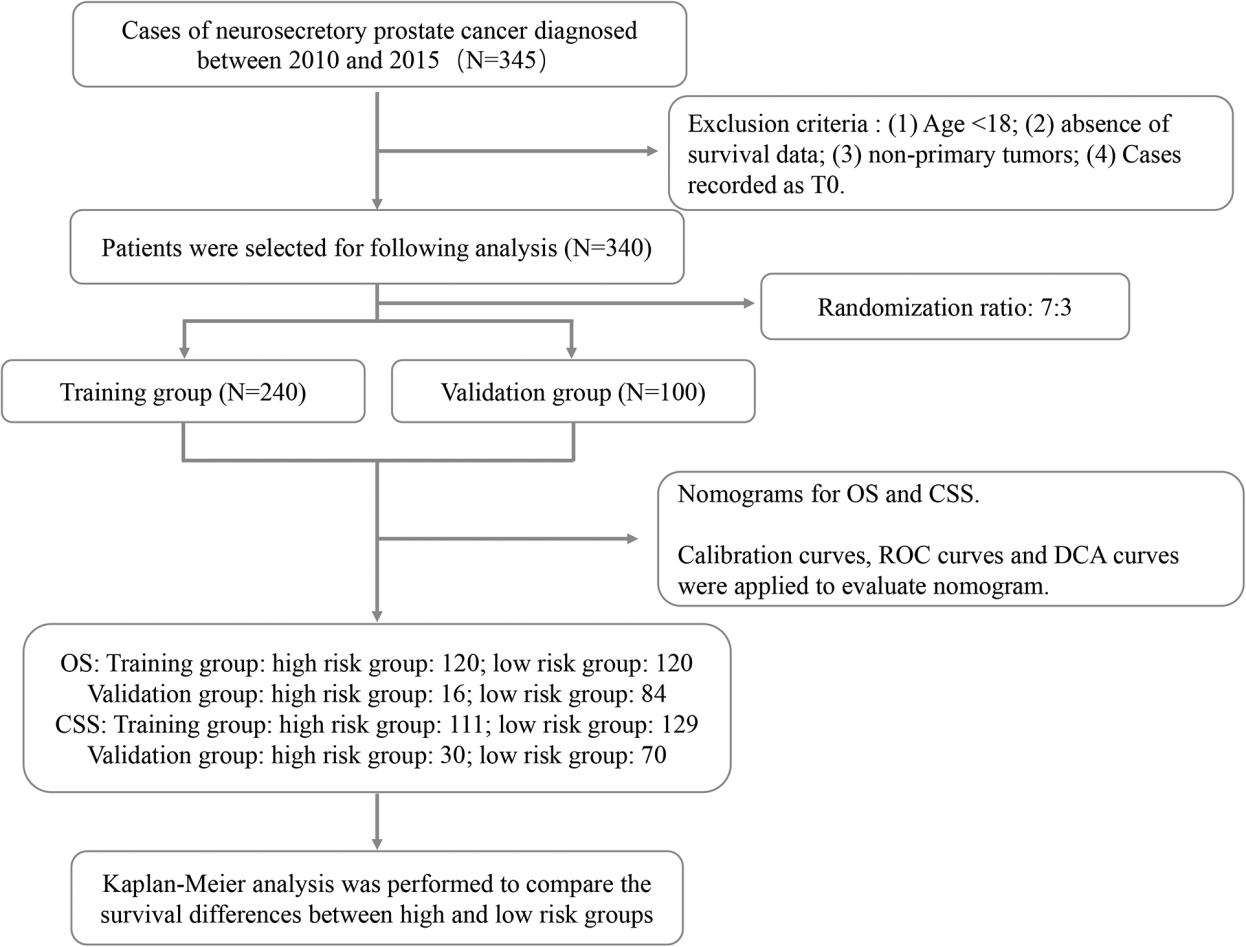
The flow chart of patient selection and data analysis.

340 patients were chosen from the SEER database. We randomly selected 240 patients as the training group, and the remaining 100 cases were selected to be included in the validation group (7:3 ratio). The details of patient selection were summarized in Figure 1. The Clinicopathological date of 340 cases of NEPC are shown in Table 1. The majority of patients in the training and validation cohorts were white (77.5% and 84.0%, respectively) and married (64.2% and 66.0%, respectively). In addition, the majority of patients in both cohorts were in AJCC stage III–IV (78.3% and 79.0%, respectively). Among the training set, 98 (40.8%) patients aged < 69 years, 111 (46.3%) patients were among 69–83 years, 31 (12.9%) patients aged > 83 years. In validation group, 27 (27.0%) patients with liver metastasis, 3 (3.0%) patients with brain metastasis, 52 (52.0%) patients received chemotherapy. All variables were not statistically significantly different between the training and validation set.

**Table 1:** The clinicopathological features of the patients in training cohort and validation cohort.

Variables	Training cohort (*n* = 240), *n* (%)	Validation cohort (*n* = 100), *n* (%)	*P* value
Race			0.329
White	186 (77.5%)	84 (84.0%)	
Black	32 (13.3%)	11 (11.0%)	
Other	22 (9.2%)	5 (5.0%)	
Age (year)			0.462
<69	98 (40.8%)	34 (34.0%)	
69–83	111 (46.3%)	50 (50.0%)	
>83	31 (12.9%)	16 (16.0%)	
Marital status			0.877
Married	154 (64.2%)	66 (66.0%)	
Unmarried	71 (29.6%)	27 (27.0)	
Unknown	15 (6.3%)	7 (7.0)	
AJCC stage			0.777
I–II	25 (10.4%)	12 (12.0%)	
III–IV	188 (78.3%)	79 (79.0%)	
Unknown	27 (11.3%)	9 (9.0%)	
T stage			0.852
T1–T2	83 (34.6%)	32 (32.0%)	
T3–T4	96 (40.0%)	40 (40.0%)	
Tx	61 (25.4%)	28 (28.0%)	
N stage			0.163
N0	91 (37.9%)	49 (49.0%)	
N1	96 (40.0%)	32 (32.0%)	
Nx	53 (22.1%)	19 (19.0%)	
M stage			0.753
M0	98 (40.8%)	39 (39.0%)	
M1	142 (59.2%)	61 (61.0%)	
Surgery			0.706
Yes	77 (32.1%)	30 (30.0%)	
No/Unknown	163 (67.9%)	70 (70.0%)	
Radiotherapy			0.550
Yes	69 (28.8%)	32 (32.0%)	
No/Unknown	171 (71.2%)	68 (68.0%)	
Chemotherapy			0.989
Yes	125 (52.1%)	52 (52.0%)	
No/Unknown	115 (47.9%)	48 (48.0%)	
Bone metastasis			0.628
No	135 (56.3%)	54 (54.0%)	
Yes	80 (33.3%)	38 (38.0%)	
Unknown	25 (10.4%)	8 (8.0%)	
Brain metastasis			0.602
No	207 (86.3%)	90 (90.0%)	
Yes	8 (3.3%)	3 (3.0%)	
Unknown	25 (10.4%)	7 (7.0%)	
Liver metastasis			0.415
No	165 (68.8%)	65 (65.0%)	
Yes	50 (20.8%)	27 (27.0%)	
Unknown	25 (10.4%)	8 (8.0%)	
Lung metastasis			0.827
No	176 (73.3%)	75(75.0%)	
Yes	37(15.4%)	16(16.0%)	
Unknown	27(11.3%)	9(9.0%)	

### The risk factors of OS and CSS for NEPC

The univariate analysis indicated that race, age, AJCC stage, N or M stage, liver or lung metastasis were closely correlated with OS and CSS, and radiotherapy was associated with OS (*P* < 0.05) (Tables 2, 3). Further analysis showed that race, age, AJCC stage, T stage, chemotherapy, and liver metastasis were related to OS in the multivariate analysis (Table 2). In addition, six risk factors, including race, age, T stage, chemotherapy, brain metastasis, and liver metastasis, were closely associated with CSS (*P* < 0.05) (Table 3).

**Table 2:** Univariate and multivariate analyses of OS risk factors for NEPC.

Variables	Univariate analysis		Multivariate analysis	
	HR (95% CI)	*P* value	HR (95% CI)	*P* value
Race		0.078		0.090
White	Reference		Reference	
Black	1.027 (0.692–1.524)	0.896	0.935 (0.606–1.443)	0.761
Other	1.694 (1.071–2.678)	0.024	2.075 (1.278–3.369)	0.003
Age (year)		<0.001		<0.001
<69	Reference		Reference	
69–83	1.481 (1.104–1.986)	0.009	1.551 (1.118–2.152)	0.009
>83	2.259 (1.480–3.447)	<0.001	2.803 (1.709–4.596)	<0.001
Marital status		0.933		0.799
Married	Reference		Reference	
Unmarried	0.970 (0.720–1.307)	0.842	1.031 (0.744–1.428)	0.855
Unknown	1.081 (0.623–1.874)	0.782	1.251 (0.645–2.429)	0.507
AJCC stage		0.032		0.084
I–II	Reference		Reference	
III–IV	1.915 (1.172–3.128)	0.009	2.090 (1.066–4.097)	0.032
Unknown	1.644 (0.890–3.036)	0.112	1.303 (0.562–3.021)	0.537
T stage		0.791		0.013
T1–T2	Reference		Reference	
T3–T4	1.100 (0.804–1.504)	0.551	0.781 (0.543–1.122)	0.181
Tx	1.111 (0.782–1.579)	0.558	0.505 (0.320–0.797)	0.003
N stage		0.016		0.236
N0	Reference		Reference	
N1	1.513 (1.110–2.061)	0.009	1.363 (0.954–1.948)	0.089
Nx	1.521 (1.059–2.184)	0.023	1.208 (0.721–2.024)	0.473
M stage				
M0	Reference		Reference	
M1	1.468 (1.110–1.942)	0.007	0.822 (0.497–1.359)	0.445
Surgery				
Yes	Reference		Reference	
No/Unknown	0.988 (0.743–1.314)	0.934	0.872 (0.642–1.185)	0.382
Radiotherapy				
Yes	Reference		Reference	
No/Unknown	1.366 (1.013–1.842)	0.041	1.139 (0.810–1.601)	0.455
Chemotherapy				
Yes	Reference		Reference	
No/Unknown	1.271 (0.969–1.666)	0.083	1.624 (1.153–2.286)	0.005
Bone metastasis		0.171		0.370
No	Reference		Reference	
Yes	1.227 (0.917–1.641)	0.169	1.306 (0.895–1.906)	0.239
Unknown	1.434 (0.922–2.230)	0.110	0.942 (0.346–2.806)	0.919
Brain metastasis		0.142		0.218
No	Reference		Reference	
Yes	1.653 (0.812–3.365)	0.165	1.918 (0.892–4.122)	0.095
Unknown	1.389 (0.906–2.128)	0.132	0.570 (0.062–5.245)	0.620
Liver metastasis		<0.001		<0.001
No	Reference		Reference	
Yes	3.200 (2.259–4.534)	<0.001	3.738 (2.415–5.787)	<0.001
Unknown	1.702 (1.102–2.630)	0.017	2.528 (0.557–11.464)	0.229
Lung metastasis		0.024		0.883
No	Reference		Reference	
Yes	1.572 (1.092–2.262)	0.015	1.024 (0.667–1.570)	0.915
Unknown	1.414 (0.932–2.146)	0.103	1.439(0.335–6.177)	0.625

**Table 3:** Univariate and multivariate analyses of CSS risk factors for NEPC.

Variables	Univariate analysis		Multivariate analysis	
	HR (95% CI)	*P* value	HR (95% CI)	*P* value
Race		0.035		0.002
White	Reference		Reference	
Black	0.863 (0.551–1.350)	0.518	0.812 (0.499–1.321)	0.402
Other	1.788 (1.116–2.865)	0.016	2.320 (1.409–3.818)	0.001
Age (year)		0.001		<0.001
<69	Reference		Reference	
69-83	1.440 (1.055–1.966)	0.022	1.510 (1.066–2.138)	0.020
>83	2.261 (1.448–3.532)	<0.001	2.851 (1.689–4.815)	<0.001
Marital status		0.918		0.846
Married	Reference		Reference	
Unmarried	0.941 (0.685–1.292)	0.705	1.006 (0.710–1.427)	0.972
Unknown	1.032 (0.570–1.868)	0.917	1.233 (0.606–2.512)	0.563
AJCC stage		0.036		0.186
I–II	Reference		Reference	
III–IV	1.937 (1.151–3.261)	0.013	1.867 (0.912–3.823)	0.088
Unknown	1.544 (0.798–2.987)	0.197	1.166 (0.473–2.875)	0.738
T stage		0.341		0.062
T1–T2	Reference		Reference	
T3–T4	1.283 (0.918–1.792)	0.144	0.938 (0.633–1.391)	0.750
Tx	1.186 (0.810–1.737)	0.381	0.576 (0.352–0.944)	0.029
N stage		0.042		0.495
N0	Reference		Reference	
N1	1.442 (1.038–2.002)	0.029	1.253 (0.858–1.828)	0.243
Nx	1.509 (1.030–2.208)	0.034	1.201 (0.702–2.055)	0.503
M stage				
M0	Reference		Reference	
M1	1.503 (1.116–2.024)	0.007	0.826 (0.484–1.412)	0.485
Surgery				
Yes	Reference		Reference	
No/Unknown	0.994 (0.735–1.344)	0.967	0.857 (0.618–1.189)	0.357
Radiotherapy				
Yes	Reference		Reference	
No/Unknown	1.305 (0.953–1.787)	0.096	1.120 (0.782–1.604)	0.537
Chemotherapy				
Yes	Reference		Reference	
No/Unknown	1.200 (0.900–1.600)	0.214	1.535 (1.066–2.209)	0.021
Bone metastasis		0.304		0.188
No	Reference		Reference	
Yes	1.246 (0.917–1.693)	0.160	1.293 (0.866–1.928)	0.209
Unknown	1.276 (0.781–2.086)	0.330	0.496 (0.152–1.611)	0.243
Brain metastasis		0.100		0.116
No	Reference		Reference	
Yes	1.881 (0.922–3.837)	0.082	2.253 (1.041–4.874)	0.039
Unknown	1.382 (0.876–2.181)	0.164	0.792 (0.091–6.882)	0.833
Liver metastasis		<0.001		<0.001
No	Reference		Reference	
Yes	3.365 (2.333–4.853)	<0.001	3.982 (2.502–6.337)	<0.001
Unknown	1.706 (1.072–2.716)	0.024	2.676 (0.614–11.668)	0.190
Lung metastasis		0.022		0.732
No	Reference		Reference	
Yes	1.629 (1.112–2.386)	0.012	1.059 (0.674–1.664)	0.803
Unknown	1.427 (0.916–2.223)	0.116	1.757(0.405–7.618)	0.451

### Construction of a prognostic nomogram for OS and CSS for NEPC

**Figure 2:**
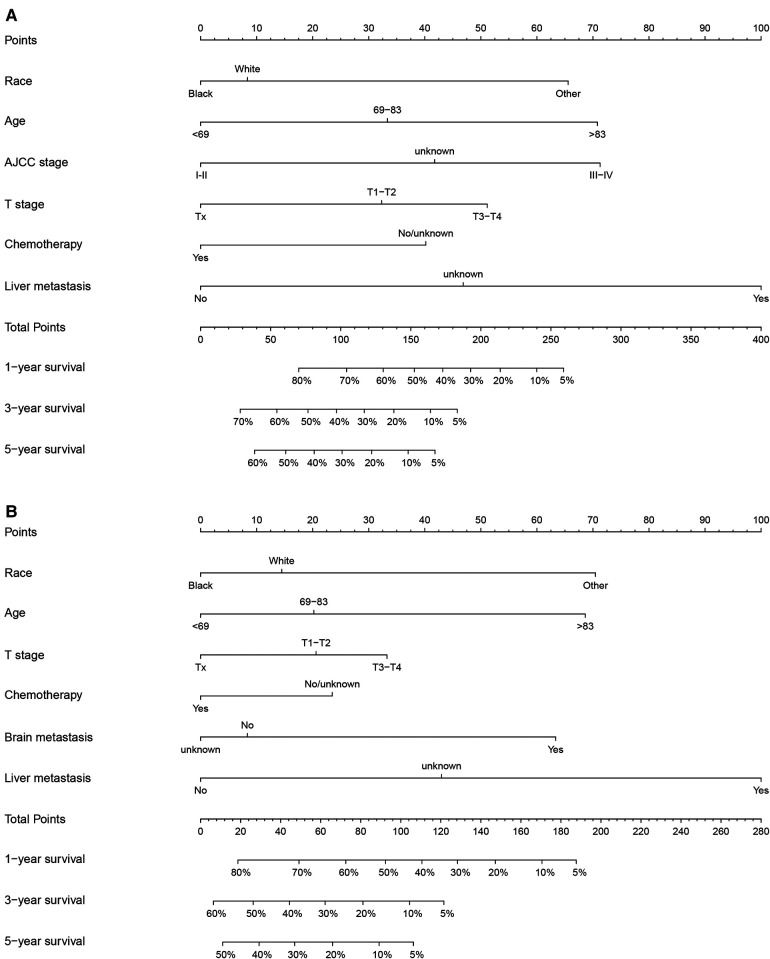
Nomograms to predict 1-, 3-, and 5-year OS or CSS of NEPC patients. (**A**) Nomogram for OS; (**B**) Nomogram for CSS.

We constructed nomograms of 1-, 3-, and 5-year OS (Figure 2A) and CSS (Figure 2B) using risk factors determined by multivariate analysis. Each risk factor corresponds to the value on the points scale, and all the risk factor points are added up to calculate the corresponding points. By drawing a line perpendicular to the total score axis, 1-, 3-, and 5-year probability of OS or CSS can be predicted for a single patient.

### Verification of the accuracy of the prognostic nomogram for OS and CSS

**Figure 3:**
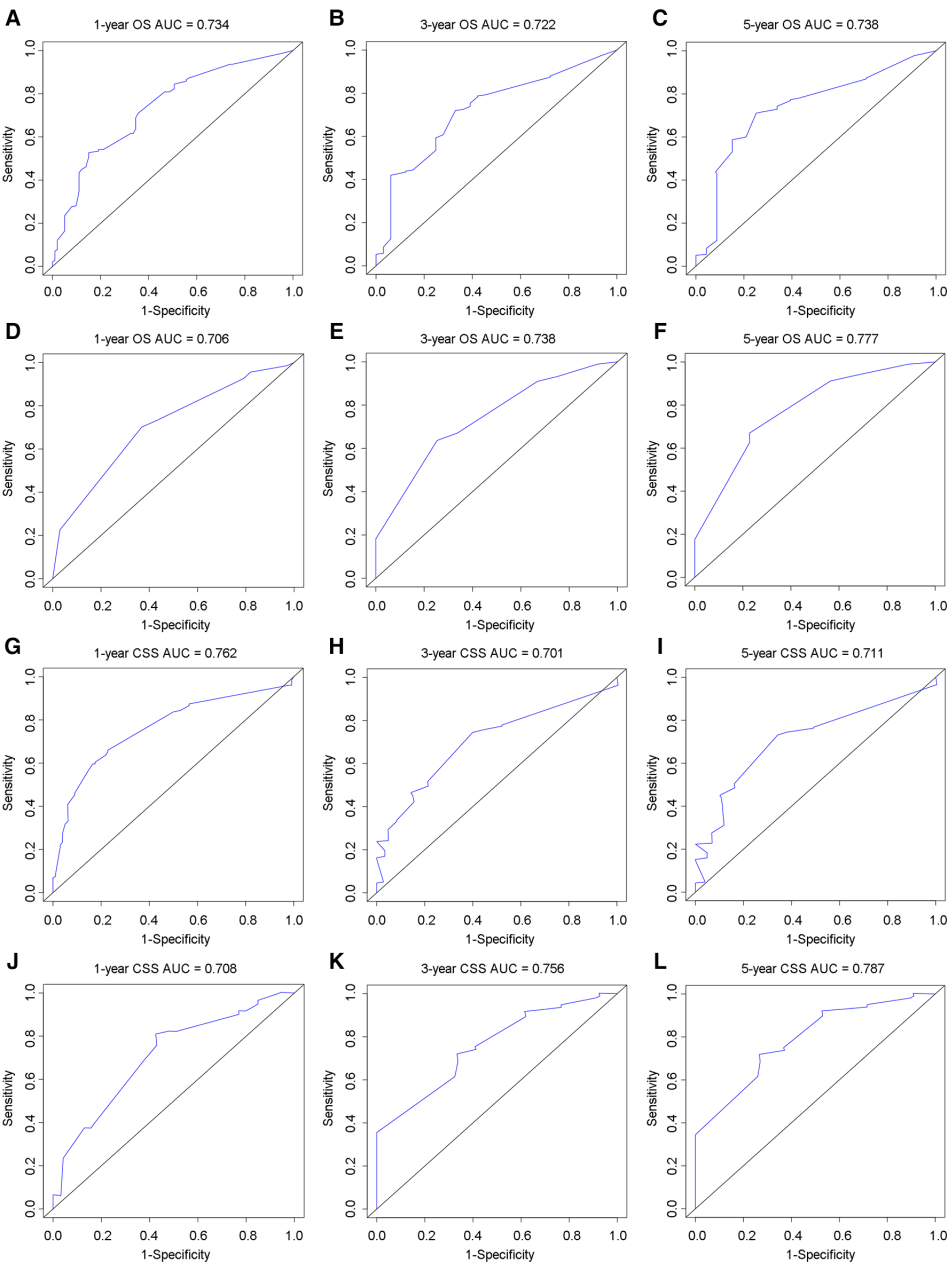
ROC curves to analyze the accuracy of the nomogram for 1-, 3-, and 5-year OS and CSS. ROC for OS at 1-year (**A**), 3-year (**B**), 5-year (**C**) in training group; ROC for OS at 1-year (**D**), 3-year (**E**), 5-year (**F**) in validation group; ROC for CSS at 1-year (**G**), 3-year (**H**), 5-year (**I**) in training group; ROC for CSS at 1-year (**J**), 3-year (**K**), 5-year (**L**) in validation group.

**Figure 4:**
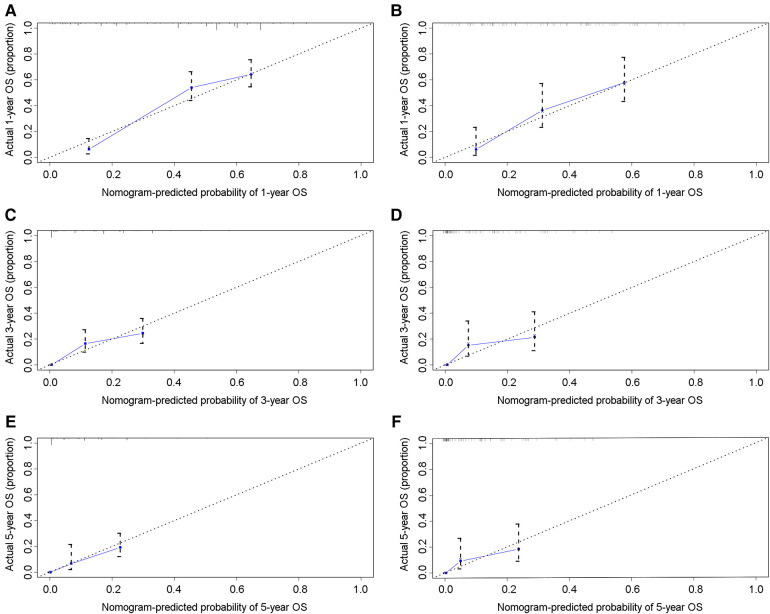
Calibration curves of the 1-, 3-, and 5-year OS of NEPC patients. 1-year (**A**), 3-year (**C**), 5-year (**E**) OS in training group; 1-year (**B**), 3-year (**D**), 5-year (**F**) OS in validation group.

**Figure 5:**
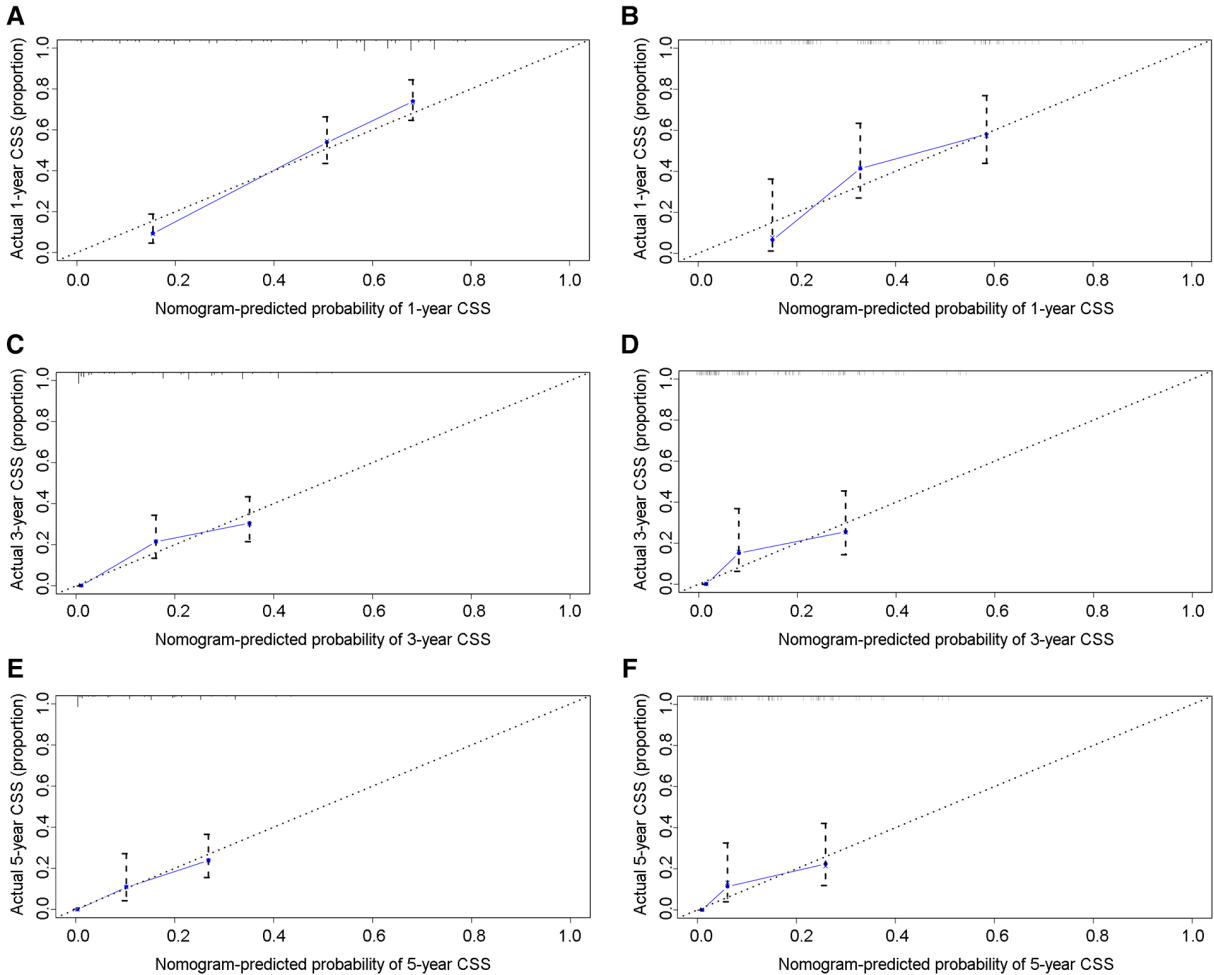
Calibration curves of the 1-, 3-, and 5-year CSS of NEPC patients. 1-year (**A**), 3-year (**C**), 5-year (**E**) CSS in training group; 1-year (**B**), 3-year (**D**), 5-year (**F**) CSS in validation group.

**Figure 6:**
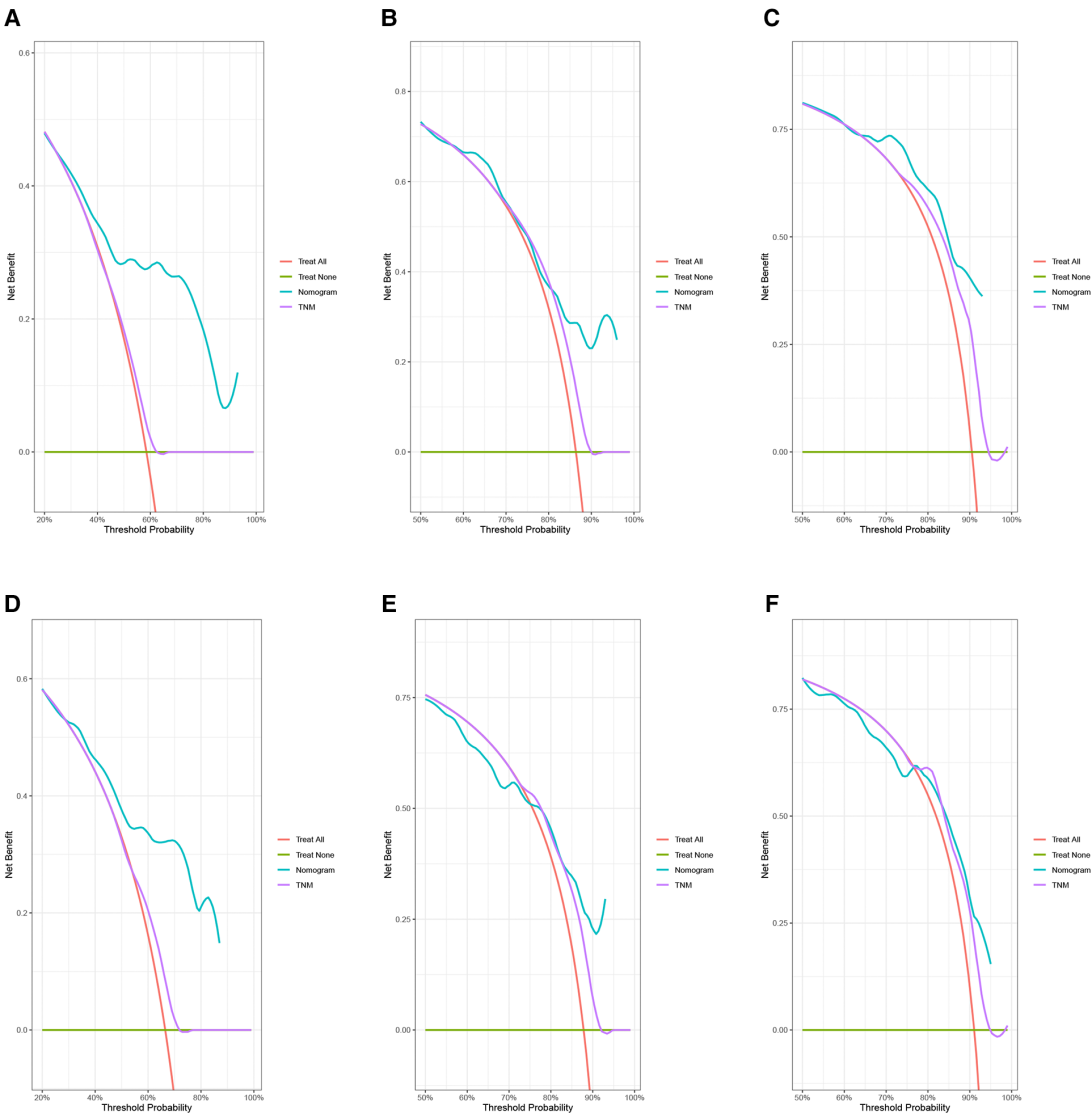
DCA curves of the 1-, 3-, and 5-year OS of NEPC patients. 1-year (**A**), 3-year (**B**), 5-year (**C**) OS in training group; 1-year (**D**), 3-year (**E**), 5-year (**F**) OS in validation group.

**Figure 7:**
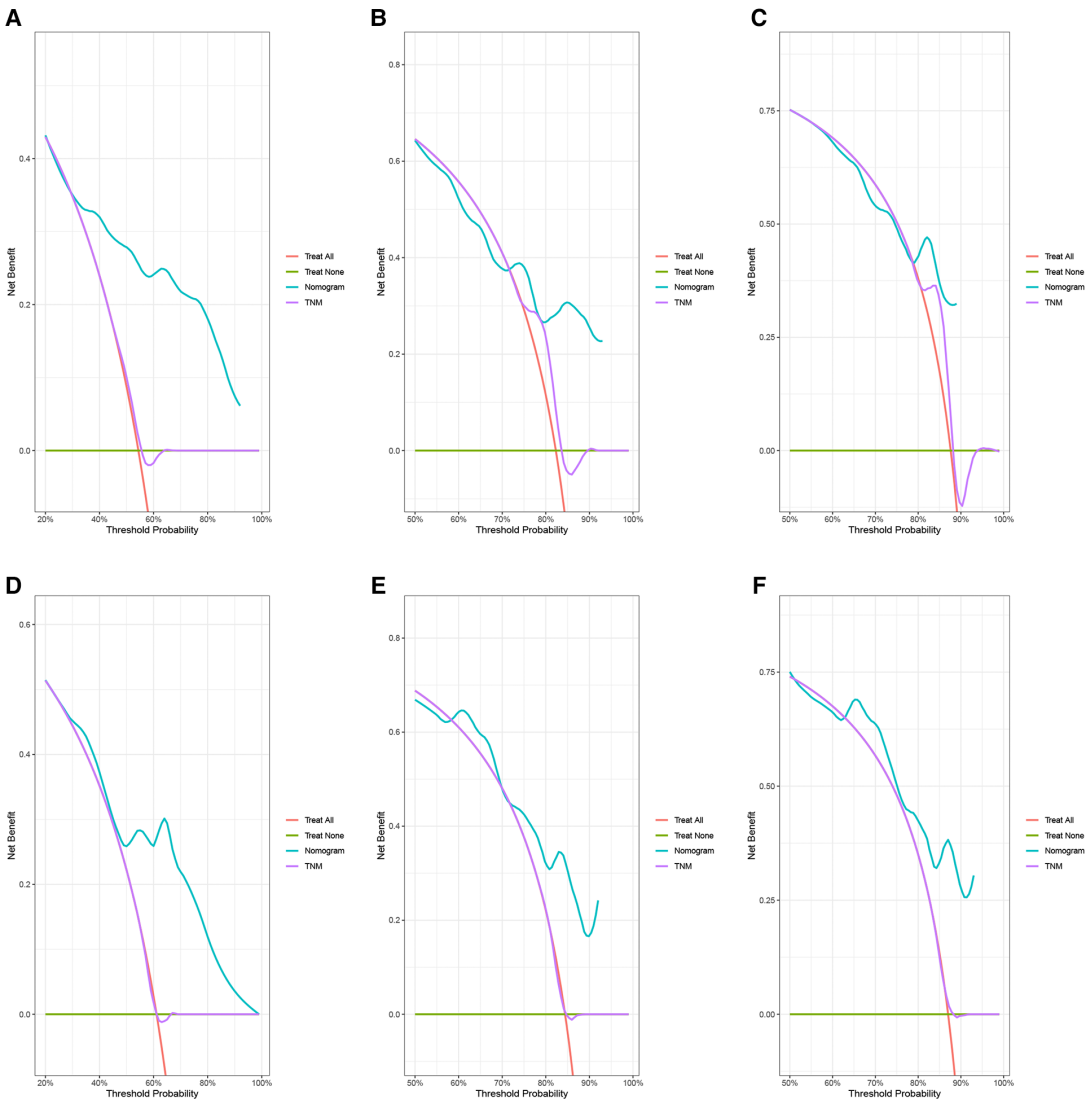
DCA curves of the 1-, 3-, and 5-year CSS of NEPC patients. 1-year (**A**), 3-year (**B**), 5-year (**C**) CSS in training group; 1-year (**D**), 3-year (**E**), 5-year (**F**) CSS in validation group.

In the training group, the C-index for OS was 0.684 (95%CI: 0.648–0.721). After calculation, the C-index was 0.689 (95%CI: 0.654–0.725) for the CSS. This meant that the nomograms we constructed had high prediction accuracy. In the validation group, the C-index for OS and CSS were 0.702 (95%CI: 0.647–0.757) and 0.686 (95%CI: 0.624–0.748), respectively. Moreover, the ROC curves also showed the models had good prediction accuracy whether in the training set or the validation set (Figure 3). For the OS, the AUCs for the 1-, 3-, and 5-year survival were 0.734 (Figure 3A), 0.722 (Figure 3B) and 0.738 (Figure 3C) in the training cohort. Beyond this, in the validation group, the AUCs for the 1-, 3-, and 5-year survival were 0.706 (Figure 3D), 0.738 (Figure 3E) and 0.777 (Figure 3F). Similar to the OS, the model showed a good accuracy to predict CSS in both training group (1-,3-, and 5-years AUC: 0.762, 0.701, and 0.711) (Figures 3G–I) or the validation group (1-,3-, and 5-years AUC: 0.708, 0.756 and 0.787) (Figures 3J–L) over a period of 5 years. To go a step further, the calibration curves were established to assess the agreement between the estimated value of the nomogram and the actual value. This result showed that no matter in training cohort (Figures 4A,C,E) or validation cohort (Figures 4B,D,F), the estimated value of the nomogram and the actual value showed excellent consistency in the OS of 1-, 3-, and 5-year. And this consistency was also well reflected in the training set (Figures 5A,C,E) and the validation set (Figures 5B,D,F) of CSS. In addition, the DCA curve in (Figures 6, 7) showed that the established NEPC nomogram has better clinical applicability than the classic TNM tumor staging.

### Predict the probability of OS or CSS by risk

**Figure 8:**
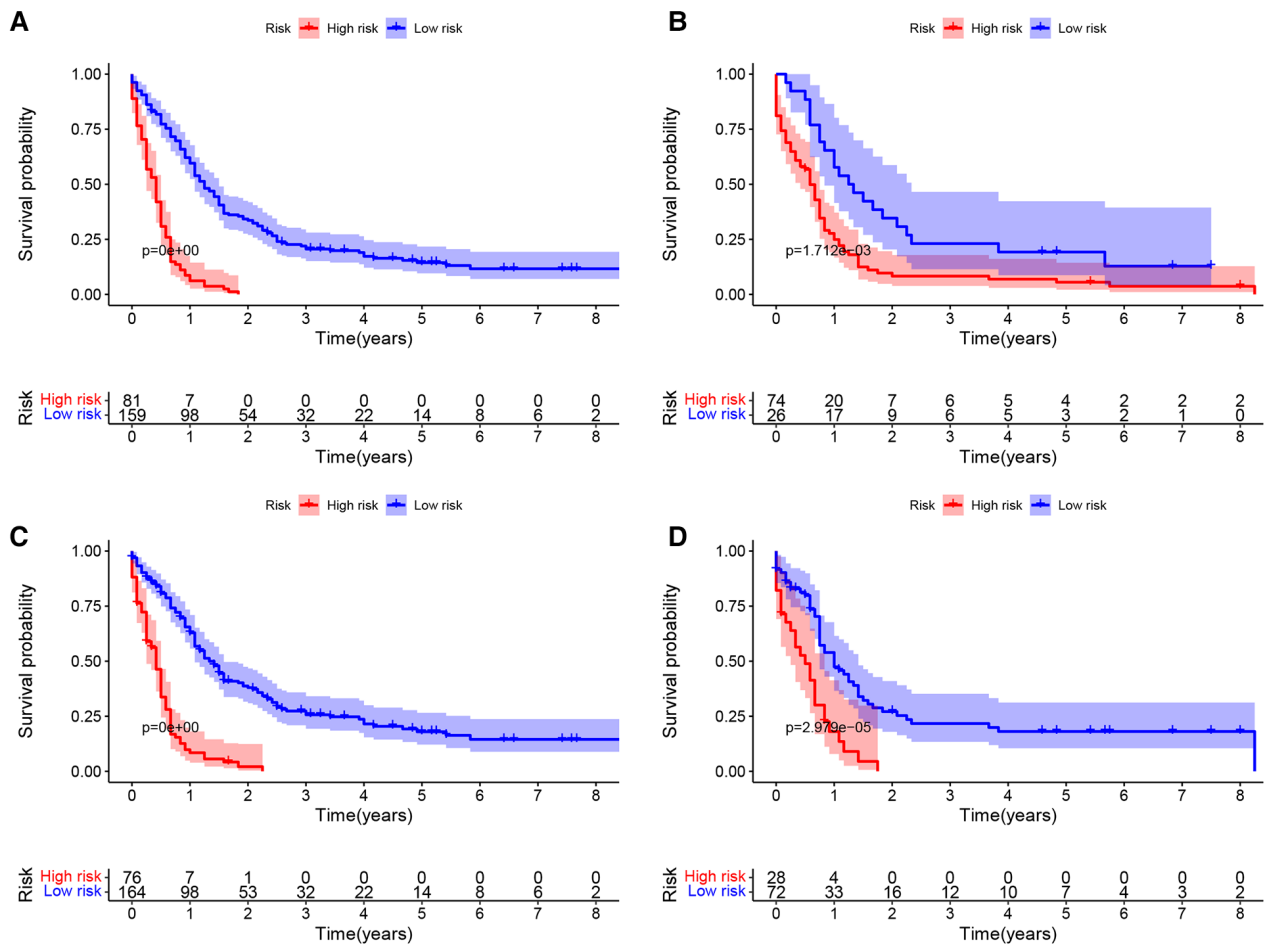
Kaplan–meier curves of OS and CSS of NEPC patients by different risk levels. OS in training set (**A**) and validation set (**B**); CSS in training set (**C**) and validation set (**D**).

Based on the nomogram, the patients were divided into two groups according to the degree of risk based on the threshold values obtained by the X-tile software. For OS, patients were divided into low-risk group (total score <193.28) and high-risk group (total score ≥193.28). For CSS, patients were divided into low risk group (total score <129.92) and high risk group (total score ≥129.92). In training cohort, by analyzing the survival curve generated by Kaplan-Meier survival analysis, it was indicated that the prognosis of the two groups was significantly different in OS or CSS (Figures 8A,C). In the validation cohort, the results further demonstrated significant differences in the prognosis of OS or CSS between patients in the low-risk group and the high-risk group (Figures 8B,D).

## Discussion

At present, medical data mining is more and more applied in clinical practice (9). Clinical big data plays an important role in the establishment of prognostic models, assessment of risk factors, diagnosis and treatment of diseases, and benefits patients a lot (10, 11). Today, the nomogram has become an important decision-making tool in modern medicine to predict disease risk or long-term survival outcomes (12, 13). The nomogram visualizes the probability of disease or death for each patient by quantifying the impact of various individual risk factors on the outcome event (14, 15). Given that NEPC is a rare and highly aggressive malignancy, the prognosis of patients with NEPC remains a challenging issue for physicians, yet most NEPC investigations are based on case reports or retrospective studies limited by small sample sizes (16–20). Therefore, this study examined a cohort of prostate cancer patients based on a large population from the SEER registry between 2010 and 2015. We aim to investigate the prognostic value of NEPC based on various clinicopathological features and construct a survival prognosis nomogram.

Various factors such as age, race, marital status, and TNM stage have been shown to be independent prognostic factors for prostate cancer (21, 22). In contrast to their study, we developed a prognostic model for NEPC patients only, which is essential to exclude the heterogeneity of common prostate cancer and NEPC. In our study, race, age, AJCC stage, T stage, chemotherapy, and liver metastasis were strongly associated with OS, whereas race, age, T stage, chemotherapy, brain metastasis, and liver metastasis were independent prognostic factors for CSS. Interestingly, radiotherapy was not correlated with OS or CSS in NEPC. Age and race have long been identified as predictors of survival in prostate cancer patients (23, 24). As age increases, the risk of death is higher. Surprisingly, we found that other ethnic groups such as American Indians /AK Natives and Asian/Pacific Islanders had worse survival probabilities compared to whites, contrary to the conclusion of the general prostate cancer study(25, 26), but consistent with the results of other NEPC studies (27). Akoto et al. noted that most deaths associated with PCa are caused by metastatic disease characterized by prostate tumor cells metastasizing to various distant organs (28). Similarly, we found that NEPC patients with liver or brain metastases had a higher risk of death, especially for liver metastases. Regarding clinical staging, they are widely considered to be robust prognostic predictors and are used in combination to analyze the risk of death in patients with NEPC (29).

The TNM staging system is considered the standard method for staging prostate patients (30). However, the inherent limitations of the TNM staging system are unavoidable, as it only emphasizes the primary tumor site, regional lymph node involvement, and distant metastasis in assessing patient prognosis, without considering other factors that affect patient prognosis, such as age, race, surgery, and chemoradiotherapy (31, 32). The advantage of establishing prognostic assessment tools is that they provide intuitive initial survival expectations on which clinicians and patients can jointly determine treatment options. However, predictive tools are not a substitute for clinical judgment, and clinicians need to make trade-offs based on individual differences, such as severity of comorbidities and physical conditions. This is the first column graph to evaluate survival in patients with NEPC and may therefore provide new clues for clinical transformation.

Our study had some limitations. First, the incidence of NEPC is too low and easy to be ignored, resulting in only 340 eligible cases in the database we eventually included. In addition, the low incidence made it difficult for us to collect enough external cases for external verification, so we could only use internal verification. Secondly, since our study was a retrospective case study based on the SEER database, related prognostic factors such as PSA and Gleason score for prostate cancer were not available. However, we have included basic variables such as age, stage, metastatic status and other important prognostic factors and will not cause devastating deviations. Lastly, as a retrospective study, the model we have established still needed some prospective clinical trials to verify it.

## Conclusion

In conclusion, in our research, we identified the risk factors of NEPC through univariate and multivariate analyses and constructed the nomogram of OS and CSS for 1-, 3-, and 5-year. We further conducted ROC analysis and established calibration curves and DCA curves to verify the accuracy of the nomogram of OS and CSS. To the best of our knowledge, our study applied a nomogram on the basis of the SEER database to NEPC for the first time. Therefore, the nomogram has the potential to be broadly applied to NEPC patients for specialized individual situation assessment and clinical decision-making.

## Data Availability

The original contributions presented in the study are included in the article/Supplementary Material, further inquiries can be directed to the corresponding author/s.
